# Impulsivity and Alcohol-Drinking Behavior: Evidence from Japan

**DOI:** 10.3390/bs13050391

**Published:** 2023-05-08

**Authors:** Sayaka Fukuda, Takuya Katauke, Saki Hattori, Soma Tanaka, Yu Kurushima, Yoichi Arakawa, Nao Ikeda, Haruka Kinoshita, Mikito Urayama, Ryota Shimizu, Tomohide Anan, Shinya Ifuku, Yuta Shiwaku, Mostafa Saidur Rahim Khan, Yoshihiko Kadoya

**Affiliations:** 1 School of Economics, Hiroshima University, 1-2-1 Kagamiyama, Higashihiroshima 7398525, Japan; m232090@hiroshima-u.ac.jp (S.F.); m235713@hiroshima-u.ac.jp (T.K.); b190428@hiroshima-u.ac.jp (S.H.); b191093@hiroshima-u.ac.jp (S.T.); b192908@hiroshima-u.ac.jp (Y.K.); b191678@hiroshima-u.ac.jp (Y.A.); b194593@hiroshima-u.ac.jp (N.I.); b191642@hiroshima-u.ac.jp (H.K.); b192737@hiroshima-u.ac.jp (M.U.); b193028@hiroshima-u.ac.jp (R.S.); b195116@hiroshima-u.ac.jp (T.A.); b191551@hiroshima-u.ac.jp (S.I.); ykadoya@hiroshima-u.ac.jp (Y.K.); 2 Kagoshima Corporate Business Office, Sumitomo Mitsui Banking Corporation, 1-38 Higashisengoku-cho, Kagoshima City 8920842, Japan; yuta.shiwaku@gmail.com

**Keywords:** alcohol, drinking, impulsivity, hyperbolic discounting, sign effect, procrastination

## Abstract

Despite the attempt by the Japanese government to reduce alcohol consumption, reduction of alcohol consumption requires improvement. We explore this issue from the impulsivity perspective and investigate whether a causal relationship exists between impulsivity and drinking behavior. We used data from the Preference Parameter Study of Osaka University to capture respondents’ drinking status. Our probit regression showed that procrastination, a proxy measure of impulsivity, was significantly associated with drinking behavior, while hyperbolic discounting, a direct measure of impulsivity, was insignificant. Our findings suggest that impulsive people will discount their health in the future; thus, the government should consider impulsivity in policymaking. For example, awareness programs should focus more on future healthcare costs from alcohol-related problems so that impulsive drinkers can understand how much they may need to spend in the future compared to current satisfaction with alcohol drinking.

## 1. Introduction

Alcohol consumption has been an important public issue in Japan for several decades [1]. Heavy drinking has adverse effects on people’s health and mortality, and can result in different types of injury [2,3,4]. To reduce heavy drinking, the Japanese government set recommended alcohol consumption limits (males > 40 g or approximately 3 cans of beer (350 mL × 3) per day, females > 20 g or approximately 1.5 cans of beer (350 mL × 1.5) per day) and promoted “The second term of the National Health Promotion Movement in the twenty-first century (Health Japan 21 [the second term])”—a national health promotion program—from 2013 to 2022 [5]. However, despite the government program, alcohol consumption has increased, especially among females [6], possibly due to their increasing participation in formal labor market [7]. We believe that government policies to reduce alcohol consumption have room for improvement.

To solve this important public issue, investigating people’s psychology of drinking could be rewarding. There is empirical evidence that suggests that people’s drinking status depends on their impulsivity or strong present-bias, which makes people myopic about future rewards [8,9,10,11,12]; however, these studies have at least three weaknesses. First, they may suffer from sample selection bias. The scope of some studies, such as Field et al. [8] and Vuchinich and Simpson [11], mainly concerns university or college students, yet sociocultural and socioeconomic characteristics also affect people’s drinking behavior [13]. Thus, comprehensive analyses of various demographic and socioeconomic characteristics are required. Second, some studies have suffered from limited sample size. For example, Petry [9] and Mitchell et al. [10] use 46 and 28 respondents, respectively. So, studies with larger sample sizes are needed. Third, most of the studies have only used delay discounting (or hyperbolic discounting indicating that a person has less patience in the immediate future compared to the distant future) to measure impulsivity via monetary choice questions, yet individuals’ time discount rates may vary depending on the sign of the reward (positive sign indicates gain and negative sign indicates loss) [14]. Accordingly, we should control for the sign effect, indicating a comparison of the time discount rates during the loss and gain phases. The sign effect generally indicates that a person discounts positive outcomes more intensely than negative payoffs. Although Sheffer et al. [12] conduct a comprehensive study in terms of scope and sample size, their analysis does not consider the sign effect. Regarding other health risk behaviors, Kang and Ikeda [15] and Ikeda et al. [16] examine the association between hyperbolic discounting and smoking behavior and hyperbolic discounting and body mass index (BMI), respectively, to control for the sign effect. However, to the best of our knowledge, no study has examined the effect of hyperbolic discounting on drinking behavior by controlling for the sign effect. This is the first study to explore the relationship between impulsivity and alcohol consumption, so as to deal with the aforementioned issues in the previous studies. Specifically, our study fills the gaps related to sampling bias, sample selection bias, and controlling for the sign effect. Moreover, the use of alternative measures of impulsivity allows us to better understand the association between impulsivity and drinking behavior. We believe that our results implicate policymakers in the impulsivity perspective toward alcohol-related issues and can provide objective evidence.

## 2. Data and Methods

### 2.1. Data

We utilized data from the Preference Parameters Study (PPS) conducted by the Institute of Social and Economic Research at Osaka University. The PPS is a panel survey that has collected information on socioeconomic characteristics and preferences from a representative sample of the Japanese population since 2003. Our study utilized the 2009 wave due to the availability of data on the respondents’ drinking behavior and time discount rates—our main variables. After excluding the missing data for demographic and socioeconomic characteristics, our sample included 2992 individuals—approximately 48% of the 2009 dataset’s respondents (*N* = 6181).

### 2.2. Variables

Our dependent variable was alcohol consumption. The PPS item “Do you drink alcoholic beverages?” had six response options (1 = do not drink at all; 2 = hardly drink; 3 = drink sometimes; 4 = drink a can of beer (12 oz) or its equivalent a day, every day; 5 = drink 3 cans of beer (36 oz) or its equivalent per day, every day; and 6 = drink 5 cans of beer (60 oz) or its equivalent a day, every day). Following Putthinun et al. [1], we grouped these responses into a binary scale by coding respondents who answered one, two, or three as “zero” or “non-daily drinkers,” and those who answered four, five, or six as “one” or “daily drinkers”.

Regarding the independent variables, we calculated the respondents’ time discount rates from the responses to the five questions presented in Appendix A. Each question asked the respondents about their intertemporal choices by controlling for the magnitude of the reward and length of the delay. Following Ikeda et al. [16], each question asked the respondents to choose between two choices related to the transfer of money with different interest rates (low to high). Specifically, the interest rate increased as the respondents proceeded from the top to bottom of each response. Therefore, at a certain point, the respondents were expected to shift their choices from option “A” to option “B”. We estimated the respondents’ time discount rates based on interest rates at the switching point. Data from those who shifted more than twice were excluded from the analysis. Regarding the hyperbolic discounting, we utilized questions 6 and 7, which asked the respondents about the timing of receiving rewards in the near and distant future, respectively. After estimating each time discount rate (DR) for DR1 and DR2 for questions 6 and 7 in Appendix A, we classified the respondents as hyperbolic discounters if their DR1 rate was higher than that of DR2, and created a corresponding binary variable. Regarding the sign effect, we measured this variable using questions 9 and 10, which asked the respondents to receive a different amount 13 months from today and to pay a different amount 13 months from today, respectively. These questions were used to indicate the difference in respondents’ time discount rates related to positive and negative payoffs. Thus, we coded the respondents as one for the sign effect if DR4 > DR5 and zero if otherwise. Further, we created the impatience variable by averaging the standardized values of the elicited discount rates created by the five questions. We also created the procrastination variable, a proxy measure for impulsivity, following Kang and Ikeda [15] and Ikeda et al. [16]. We controlled for other variables to make our investigation clearer, including sex, age, university degree, marital status, household members, employment status, household income, and household assets. We also added variables related to health risk behaviors, such as smoking and gambling. Finally, we controlled for risk aversion, current level of happiness, and health anxiety. Table 1 presents the variable definitions.

### 2.3. Descriptive Statistics

Table 2 presents the time discount rates under controlled conditions with the magnitude of the reward and time horizons. The mean value of impatience, which represents the standardized value of the respondents’ time discount rate from DR1–DR5, is −3.80 × 10^−9^. Moreover, the mean standardized discount rate of the respondents for the current period (DR1) is 65.79% when that of after three months (DR2) is 64.25%. There are no significant differences between DR1 and DR2 for the overall sample. Thus, hyperbolic discounting is not identified as an overall trend within the sample. However, the sign effect shows differences in that DR4 is generally higher than DR5 in the sample.

Table 3 shows the respondents’ descriptive statistics. Overall, 27.7% of the respondents are daily drinkers. Regarding the time discount factors, 13.3% are hyperbolic discounters and the sign effect is observed among 70.3%. However, the respondents rate the level of procrastination (a proxy for hyperbolic discounting) as 3.27. Regarding the demographic characteristics, 46.4% of the respondents are male, the average age is 49.7 years, 26.5% have university qualifications, 81.7% are married and 10.1% are divorced, 82.3% have at least one child, and the average number of people in the household is 3.5. Regarding the socioeconomic variables, household income and household assets account for around JPY 6.7 million (equivalent to $67,000) and JPY 13.8 million (equivalent to $138,000), respectively. About 70% of the respondents are employed, 23.5% are smokers, and 7.6% are frequent gamblers. Finally, the respondents rate their current levels of happiness and risk aversion as 6.55% and 50.61%, respectively, on average.

Table 4 shows the distribution of drinking behavior by age group. There is a statistically significant difference in drinking behavior between each age group that reaches a peak for middle-aged respondents.

Table 5 shows the differences in drinking behavior according to respondents’ demographic characteristics. Regarding the relationship between sex and alcohol consumption, 46% of males drink daily compared to 11% of females. The proportion of employed respondents who drink daily (32.19%) is higher than that of the unemployed respondents (17.24%). Regarding educational attainment (university degree), 33.3% of the respondents who have graduated from university are daily drinkers compared to 25.7% of the respondents who have not.

Table 6 shows the significant relationships between alcohol consumption and health risk behaviors. The results indicate that respondents who engage in health risk behaviors, such as gambling and smoking, tend to drink more.

### 2.4. Methodology

Many of the previous studies have suggested that time preference, such as time discount rate, hyperbolic discounting, and sign effects, impacts health risk behavior [15,16,17]. Therefore, we investigated the relationship between time preference and drinking behavior.

The knowledge of economics assumes that rational people discount the value of things exponentially with delays [18]. Technically, an outcome that has utility *A* if received immediately (*t* = 0) is valued as $A\times \delta^{t}$ if *t* periods are delayed in the future. Thus, the present time value (*V*) of receiving (*A*) at time (*t*) is given as:

$$V\left(A,\;t\right)=A\times \delta^{t}$$

where the discount rate δ represents a constant proportional decrease in value with each added delay period [19].

However, the psychology field proposes that people have a propensity for hyperbolic discounting [20]. This means that people tend to violate the exponential assumption of a constant proportional discount factor and discount rewards in the immediate future more heavily than those in the distant future [19]. If the respondents are hyperbolic discounters, then the shape of the discount function is described as:

$$V\left(A,\;t\right)=A\times \frac{1}{1+k.t}$$

where *k* is a parameter that indicates the rate at which the value is discounted.

Since our dependent variables were binary variables, we employed probit regression. We estimated the following equation:

$$Y_{i}=f\left(HD_{i},SE_{i},IP_{i},\;X_{i},\;\varepsilon_{i}\right)$$

where $Y_{i}$ indicates whether the ith respondent is a daily drinker. *IP* is the degree of the respondent’s impatience, based on the standardized time discount rate. *HD* and *SE* indicate the hyperbolic discounting and sign effect variables, respectively. *X* is a vector of individual characteristics, and ε is the error term. Our full estimated equation is as follows:

$$\begin{array}{ll} Daily\;drinking_{\;i} & \left(1=daily\;drinker\;and\;0=otherwise\right)\\ & =\;\beta_{0}\;+\;\beta_{1}impatience_{i}\;+\;\beta_{2}\;hyperbolic\_discounting_{i}\\ & +\;\beta_{3}sign\_effect_{i}+\;\beta_{4}male_{i}\;+\;\beta_{5}age_{i}\;+\;\beta_{6}age\_squared_{i}\\ & +\;\beta_{7}uni\_deree_{i}+\;\beta_{8}married_{i}\;+\;\beta_{9}divorce_{i}\\ & +\;\beta_{10}household\_num_{i}\;+\;\beta_{11}children_{i}+\;\beta_{12}unemployed_{i}\\ & +\;\beta_{13}log\_of\_hincome_{i}\;+\;\beta_{14}log\_of\_hasset_{i}\\ & +\;\beta_{15}smoking_{i}\;+\beta_{16}gambling_{i}\;+\beta_{17}risk\_aversion_{i}\\ & +\beta_{18}happiness_{i}\;+\beta_{19}health\_anxiety_{i}\;+\varepsilon_{i} \end{array}$$

## 3. Results

Table 7 shows the probit regression results. There is no significant relationship between hyperbolic discounting and drinking behavior. However, procrastination, a proxy measure for hyperbolic discounting, shows a significantly positive association. Regarding the other time discount measures, impatience, a standardized time discount rate, has no significant relationship with daily drinking after controlling for the demographic and socioeconomic variables. The sign effect has a positive relationship with daily drinking behavior.

Regarding the respondents’ demographic and socioeconomic characteristics, being male, married, and divorced have positive and significant associations with alcohol consumption at the 1% and 5% significance levels, respectively, while being unemployed has a negative association. The results further show that age has a positive association with drinking behavior, whereas age squared has a negative association with drinking behavior. This implies that age has a nonlinear relationship with drinking behavior. Regarding the health risk behaviors, smokers are more likely to be daily drinkers, whereas frequent gamblers show no significant relationship.

Further, drinking behavior shows a significant difference in some demographic characteristics, such as age and sex. Therefore, we conducted a subsample regression of the model, classified by sex and some age groups, to clarify the relationship between impulsivity and drinking behavior in more detail. Table 8 presents the probit regression results grouped by sex. There is no significant relationship between hyperbolic discounting and drinking behavior in the estimation results for the full sample regression. However, procrastination has a significant negative association with drinking behavior among males. The sign effect is positively associated among females, whereas impatience is not associated with drinking across all estimations.

Table 9 presents the results for the sample when classified by age group. Hyperbolic discounting is positively associated with daily drinking among respondents aged under 40 years, whereas procrastination has a positive association among respondents aged 41 years and over. However, neither the sign effect nor impatience has a significant relationship with drinking behavior.

Finally, we checked the robustness of results by measuring some variables differently. For example, rather than using a five-point scale, we used procrastination as a binary variable where 1 represents the answer “got it done at the last minute” to the question “Thinking about when you were a child and you were given an assignment in school, when did you usually do the assignment?” We also used the high school graduate variable as an alternative to a university degree. However, our results remain largely the same. We have not shown the results in the manuscript to save space, but they are available upon request.

## 4. Discussion

Alcohol consumption has been an important public issue in Japan for several decades [1]. We investigated this issue from the impulsivity perspective because people’s drinking behavior depends on their behavioral pattern. Overall, our results revealed that hyperbolic discounting, which is a direct measure of impulsivity, was positively but insignificantly associated with drinking behavior, whereas procrastination, which is an indirect proxy measure for impulsivity, was positively significant. Moreover, we found that the sign effect was significantly associated with drinking behavior. Impatience was initially positively significant but became insignificant after controlling for the respondents’ demographic and socioeconomic characteristics. We explain these results in terms of measurement error and sociocultural aspects, respectively. Finally, we found that some demographic, socioeconomic, health risk behavior, and psychological characteristics significantly affected drinking behavior.

Our probit regression results showed that hyperbolic discounting had an insignificant relationship with drinking behavior; this is inconsistent with the extant literature—Vuchinich and Simpson [11], Field et al. [8], Moore and Cusens [21], Stea et al. [22], and Lee and Liao [23]—that has suggested that people with higher alcohol consumption have steeper time discount rates. However, our results revealed significant associations among the younger people in the subsample analyses. We explain this limited association of impulsivity with drinking behavior in terms of the measurement error in the time discount rates. The previous research has suggested that the context and difficulty of the questions may affect people’s discount rates [15,24]. Conversely, we found that procrastination, a proxy measure for impulsivity, was significantly associated with drinking behavior in the full sample. Our results were consistent with other health risk behaviors in that the proxy measure was significant, whereas the direct measure was not [15,16]. However, the hyperbolic discounting and procrastination results were consistently insignificant among females in all models. This may be attributed to Japan’s cultural background. Generally, males drink more alcohol than females in Japan [25], and many females may not drink much alcohol, even if they are impulsive.

Further, we revealed that the sign effect was positively and significantly associated with drinking behavior in the full sample and female subsample. We believe that those showing the sign effect are unlikely to discount the loss of networking during drinking opportunities as much, and may value this more than the gain of maintaining health. Caluzzi et al. [26] claim that the link between health and alcohol consumption is complex, as drinking alcohol involves certain social and cultural aspects beyond its health effect. Accordingly, alcohol consumption may have other positive effects on social or cultural aspects, such as promoting social relationships. The Ministry of Health, Labour and Welfare [27] claims that “Alcohol, in small amounts, has the effect of relaxing the mind and increasing conversions.” So, the negative health effects of problematic drinking may be mitigated by the positive effects of drinking in terms of the social and cultural aspects involved. Finally, we found that although the degree of impatience was initially positively and significantly associated with daily drinking, it became insignificant, and the magnitude of the association was also reduced after controlling for demographic, socioeconomic, health risk behavior, and psychological characteristics. Meanwhile, Collins [13] asserts that socioeconomic status affects drinking behavior. Accordingly, the effect of impatience on alcohol consumption may have been mitigated by these factors.

Regarding the respondents’ demographic and socioeconomic characteristics, we found that being male, older (until reaching middle age), married, divorced, having children, and employment status were significantly associated with drinking behavior. Regarding the male, divorced, and employment status variables, the results were consistent with the extant literature that has reported that males consume more alcohol than females [1,2,27,28,29]. Alternatively, less alcohol consumption by females could be due to cultural reasons for which females tend not to report their drinking habits. Regarding age (until reaching middle age), although alcohol consumption increased with age up to a certain point, it declined progressively afterward [1,30]. Regarding marital status, married respondents were likely to have a positive attitude toward daily drinking. This tendency was particularly observed in females. We believe that married females experience considerable stress due to their household role [31]. So, they may increase their alcohol consumption to cope with stress. In addition, married females may be more vocal about their drinking habits, which unmarried females cannot express for cultural reasons. We also found a significant association between parental status and daily drinking behavior, especially among males and younger respondents. Our results are consistent with those of Putthinun et al. [1], Bowden et al. [32], and Paradis [33], who observe a negative association between parental status and drinking behavior. One possible explanation may be that parents use alcohol to relieve their parenting stress [34].

Regarding the health risk behavior and psychological characteristics, smokers tended to significantly consume alcohol; this is consistent with the results of the previous studies [1,35].

Our study had some limitations. First, we utilized hyperbolic discounting and procrastination as the direct and proxy measures of impulsivity, respectively. Although both measurements have been utilized in the extant literature, such as Kang and Ikeda [15] and Ikeda et al. [16], there may be some measurement errors due to self-reporting of hypothetical monetary choices. Further studies are required to clearly measure impulsivity using an actual monetary choice scenario over short and long periods or by using a more comprehensive impulsiveness measure (e.g., the Barratt impulsiveness scale). Second, due to data unavailability, we captured the respondents’ alcohol consumption based only on their drinking frequency. As Putthinun et al. [1] assert, it is important that future studies establish tools to more accurately capture drinking behavior. Third, we used the 2009 wave of the PPS because of its large sample size and abundance of data to capture impulsivity. However, an individual’s drinking behavior can change year by year. The lack of panel and recent data remains a limitation of this study. Therefore, further studies that utilize the latest data are required. Moreover, future studies should consider other addictive behavior and emotional conditions to provide robust evidence on the behavioral decision-making process. Despite these limitations, we believe that our study provides important implications regarding the association between impulsivity and drinking behavior.

## 5. Conclusions

We examined the causal relationship between impulsivity and alcohol consumption behavior among the Japanese population. Our probit regression results showed that procrastination, a proxy for impulsivity, was associated with drinking behavior. However, hyperbolic discounting, a direct measure of impulsivity, was not associated with drinking behavior. Further, we found that demographic, socioeconomic, health risk behavior, and psychological characteristics were associated with drinking behavior. We suggest that sociocultural and socioeconomic characteristics may mitigate the effect of impulsivity on drinking behavior.

We believe that our results make the following contributions to the literature. First, our study is the first to address sampling bias and sample selection bias by utilizing large-scale data (*N* = 2993) that include a wide range of demographic and socioeconomic characteristics. Second, our study is the first attempt to examine the relationship between impulsivity and drinking behavior by controlling for the sign effect. Our results may assist policymakers by providing a preliminary idea on how impulsivity is associated with alcohol-drinking behavior, which they can utilize in designing policies to control alcohol consumption in Japan, particularly among specific socioeconomic groups.

## Figures and Tables

**Table 1:** Variable definitions.

Variable	Definition
Dependent variable	
Drinking	A binary indicator for daily drinker: 1 = drink one/three/five cans of beer daily; 0 = do not drink at all, hardly drink, or drink sometimes
Independent variable	
Impatience	The simple mean of the standardized values of the elicited discount rates (DR1–DR5) as a measure of the degree of impatience
Hyperbolic_discounting (direct measure)	A binary indicator for hyperbolic discounting: 1 if DR1 > DR2; 0 = otherwise
Procrastination (proxy measure for hyperbolic discounting)	An ordinal indicator of a proxy measure of hyperbolic discounting or the degree of procrastination, which is the response to the following question on a five-point scale: “Thinking about when you were a child and you were given an assignment in school, when did you usually do the assignment?” (1) Got it done right away; (2) tended to get it done early, before the due date; (3) worked on it daily up until the due date; (4) tended to get it done toward the end; and (5) got it done at the last minute
Sign_effect	A binary indicator for sign effect: 1 = DR4 > DR5; 0 = otherwise
Male	Binary variable: 1 = male; 0 = female
Age	Age of respondents
Age squared	Square of age
University	Binary variable: 1 = obtained a university degree or higher; 0 = otherwise
Married	Binary variable: 1 = married; 0 = otherwise
Divorced	Binary variable: 1 = divorced; 0 = otherwise
Unemployed	Binary variable: 1 = unemployed; 0 = otherwise
Hincome	Annual earned income before taxes and with bonuses of entire household in 2008 (unit: million JPY)
Log_hincome	Natural log of hincome
Hasset	Balanced amount of financial assets (savings, stocks, insurance, etc.) of entire household in 2008 (unit: million JPY)
Log_hasset	Natural log of hasset
Household num	Number of people living in the household
Children	Binary variable: 1 = have at least one child; 0 = otherwise
Smoking	Binary variable: 1 = current smoker (sometimes more than two packs a day); 0 = otherwise (do not smoke at all, quit, or hardly smoke)
Gambling	Binary variable: 1 = frequent gambler (gamble once a week or more); 0 = otherwise
Risk aversion	Variable measuring the degree of risk aversion from the question “When you go out, how high does the probability of rainfall have to be before you take an umbrella?”
Happiness	Respondents’ current level of happiness measured by the question “Overall, how happy would you say you are currently?”
Health anxiety	Binary variable: 1 = agree or almost agree with the statement “I have anxiety about my health”; 0 = otherwise

**Table 2:** Time discount rates under controlled conditions.

		DR1	DR2	DR3	DR4	DR5	Impatience
Choice conditions	Timings (A) or (B)	2 days or 7 days	90 days or 97 days	1 month or 13 months	1 month or 13 months	1 month or 13 months	-
	Amount for (A)	JPY 10,000	JPY,10,000	JPY 10,000	JPY 1 million	JPY 1 million	-
	Receipt or payment	Receipt	Receipt	Receipt	Receipt	Payment	-
Descriptive statistics	Mean	0.6579	0.6425	0.0794	0.0134	0.0110	−3.80 × 10^−9^
	Std. dev.	0.8672	0.8720	0.0944	0.0252	0.0510	0.6555
	Obs.	2993	2993	2993	2993	2993	2993
Time discounting properties (*p*-value)		Hyperbolic discounting: DR1 > DR2 (0.1393)			Sign effect DR4 > DR5 (0.0187 **)		

Note: ** *p* < 0.05.

**Table 3:** Descriptive statistics.

Variable	Obs.	Mean	Std. Dev.	Min.	Max.
Dependent variable					
Drinking	2993	0.2769	0.4475	0	1
Main independent variables					
Hyperbolic_discounting	2993	0.1333	0.3399	0	1
Procrastination	2993	3.2746	1.3241	1	5
Sign_effect	2993	0.7026	0.4571	0	1
Impatience	2993	0.0000	0.6555	−1.3600	2.7925
Other independent variables					
Procrastination	2993	3.2746	1.3241	1	5
Male	2993	0.4637	0.4987	0	1
Age	2993	49.6595	13.0469	20	76
Age squared	2993	2636.2350	1288.4770	400	5776
University	2993	0.2649	0.4413	0	1
Married	2993	0.8165	0.3870	0	1
Divorced	2993	0.1009	0.3012	0	1
Household_num	2993	3.5205	1.4426	1	11
Children	2993	0.8225	0.3820	0	1
Unemployed	2993	0.3004	0.4585	0	1
Hincome	2993	6,659,372	3,963,443	1,000,000	20,000,000
Log_hincome	2993	15.5307	0.6269	13.8155	16.8112
Hasset	2993	13,800,000	18,400,000	2,500,000	100,000,000
Log_hasset	2993	15.8389	1.0321	14.7318	18.4206
Smoking	2993	0.2345	0.4237	0	1
Gambling	2993	0.0758	0.2647	0	1
Risk aversion	2993	0.5060	0.1933	0	1
Happiness	2993	6.5539	1.7906	0	10
Health anxiety	2993	0.4193	0.4935	0	1

**Table 4:** The distribution of drinking behavior by age group.

Drinking	Age			Total
	Under 40 Years	40–60 Years	Over 60 Years	
0	675	961	528	2164
	81.82%	67.44%	71.06%	72.30%
1	150	464	215	829
	18.18%	32.56%	28.94%	27.70%
Total	825	1425	743	2993
	100%	100%	100%	100%
F-statistic	F = 27.83 ***			

Note: *** *p* < 0.01.

**Table 5:** The distribution of drinking behavior by respondents’ demographic characteristics.

Drinking	Sex		Employed		University Degree		Total
	Female	Male	Yes	No	Yes	No	
0	1422	742	1420	744	529	1635	2164
	88.60%	53.46%	67.81%	82.76%	66.71%	74.32%	72.30%
1	183	646	674	155	264	565	829
	11.40%	46.54%	32.19%	17.24%	33.29%	25.68%	27.70%
Total	1605	1388	2094	899	793	2200	2993
	100%	100%	100%	100%	100%	100%	100%
Mean difference	*t* = −23.2745 ***		*t* = 8.4731 ***		*t* = −4.1156 ***		

Note: *** *p* < 0.01.

**Table 6:** The distribution of drinking behavior by health risk behaviors.

Drinking	Smoker		Gambler		Total
	Yes	No	Yes	No	
0	378	1786	135	2029	2164
	53.85%	77.96%	59.47%	73.36%	72.30%
1	324	505	92	737	829
	46.15%	22.04%	40.53%	26.64%	27.70%
Total	702	2291	227	2766	2993
	100%	100%	100%	100%	100%
Mean difference	*t* = −12.8240 ***		*t* = −4.5073 ***		

Note: *** *p* < 0.01.

**Table 7:** Probit regression results for drinking behavior with time discount factors.

	Dependent Variable: Daily Drinking									
	Direct Measure					Proxy Measure				
Variable	Model1	Model2	Model3	Model4	Model5	Model1	Model2	Model3	Model4	Model5
Hyperbolic_discounting	0.1055	0.0619	0.0739	0.0770	0.0733					
	(0.0708)	(0.0786)	(0.0789)	(0.0789)	(0.0792)					
Procrastination						0.0964 ***	0.0606 ***	0.0539 **	0.0534 **	0.0537 **
						(0.0190)	(0.0212)	(0.0213)	(0.0213)	(0.0213)
Sign_effect	0.0541	0.0812	0.1010 *	0.1013 *	0.0997 *	0.0615	0.0840	0.1036 *	0.1039 *	0.1022 *
	(0.0542)	(0.0591)	(0.0594)	(0.0594)	(0.0594)	(0.0543)	(0.0591)	(0.0594)	(0.0594)	(0.0594)
Impatience	0.1532 ***	0.0520	0.0524	0.0528	0.0522	0.1518 ***	0.0513	0.0524	0.0528	0.0519
	(0.0371)	(0.0411)	(0.0414)	(0.0414)	(0.0414)	(0.0371)	(0.0411)	(0.0414)	(0.0414)	(0.0414)
Male		1.1137 ***	1.0055 ***	1.0051 ***	1.0073 ***		1.0933 ***	0.9900 ***	0.9900 ***	0.9922 ***
		(0.0607)	(0.0640)	(0.0640)	(0.0641)		(0.0613)	(0.0644)	(0.0644)	(0.0645)
Age		0.0805 ***	0.0819 ***	0.0823 ***	0.0853 ***		0.0810 ***	0.0825 ***	0.0827 ***	0.0858 ***
		(0.0187)	(0.0188)	(0.0188)	(0.0190)		(0.0187)	(0.0188)	(0.0188)	(0.0190)
Age squared		−0.0007 ***	−0.0007 ***	−0.0007 ***	−0.0007 ***		−0.0007 ***	−0.0007 ***	−0.0007 ***	−0.0007 ***
		(0.0002)	(0.0002)	(0.0002)	(0.0002)		(0.0002)	(0.0002)	(0.0002)	(0.0002)
University		−0.0416	0.0089	0.0103	0.0069		−0.0228	0.0254	0.0262	0.0226
		(0.0648)	(0.0658)	(0.0659)	(0.0661)		(0.0650)	(0.0660)	(0.0661)	(0.0663)
Married		0.2829 **	0.2804 **	0.2785 **	0.2603 **		0.2874 **	0.2851 **	0.2839 **	0.2649 **
		(0.1160)	(0.1160)	(0.1161)	(0.1167)		(0.1164)	(0.1163)	(0.1163)	(0.1170)
Divorced		0.3626 ***	0.3174 ***	0.3155 ***	0.3175 ***		0.3697 ***	0.3250 ***	0.3238 ***	0.3260 ***
		(0.1086)	(0.1092)	(0.1093)	(0.1094)		(0.1091)	(0.1096)	(0.1097)	(0.1098)
Household_num		0.0226	0.0202	0.0199	0.0218		0.0214	0.0190	0.0188	0.0208
		(0.0218)	(0.0221)	(0.0221)	(0.0221)		(0.0219)	(0.0221)	(0.0221)	(0.0222)
Children		0.1704	0.1802	0.1802	0.1725		0.1931 *	0.1996 *	0.1994 *	0.1917 *
		(0.1142)	(0.1149)	(0.1149)	(0.1150)		(0.1148)	(0.1154)	(0.1154)	(0.1155)
Unemployed		−0.1859 ***	−0.1686 **	−0.1672 **	−0.1708 **		−0.1836 **	−0.1670 **	−0.1662 **	−0.1698 **
		(0.0717)	(0.0720)	(0.0720)	(0.0720)		(0.0716)	(0.0720)	(0.0720)	(0.0720)
Log_hincome		−0.0071	0.0024	0.0030	−0.0104		−0.0134	−0.0039	−0.0035	−0.0173
		(0.0519)	(0.0523)	(0.0523)	(0.0532)		(0.0518)	(0.0522)	(0.0522)	(0.0532)
Log_hasset		−0.0201	−0.0095	−0.0087	−0.0126		−0.0147	−0.0050	−0.0045	−0.0085
		(0.0304)	(0.0306)	(0.0306)	(0.0309)		(0.0304)	(0.0306)	(0.0306)	(0.0308)
Smoking			0.4201 ***	0.4169 ***	0.4270 ***			0.4103 ***	0.4085 ***	0.4190 ***
			(0.0642)	(0.0642)	(0.0648)			(0.0643)	(0.0643)	(0.0648)
Gambling			−0.0065	−0.0089	−0.0121			−0.0091	−0.0105	−0.0135
			(0.0982)	(0.0984)	(0.0985)			(0.0985)	(0.0987)	(0.0988)
Risk aversion				−0.0835	−0.0770				−0.0485	−0.0422
				(0.1389)	(0.1388)				(0.1390)	(0.1389)
Happiness					0.0215					0.0223
					(0.0166)					(0.0166)
Health anxiety					−0.0276					−0.0281
					(0.0556)					(0.0556)
Constant	−0.6472 ***	−3.3888 ***	3.8742 ***	3.8675 ***	3.8018 ***	0.9587 ***	−3.5938 ***	−4.0403 ***	−4.0339 ***	−3.9699 ***
	(0.0463)	(0.8357)	(0.8389)	(0.8390)	(0.8414)	(0.0791)	(0.8368)	(0.8408)	(0.8413)	(0.8437)
Obs.	2993	2993	2993	2993	2993	2993	2993	2993	2993	2993
Log likelihood	−1756	−1465	−1442	−1442	−1441	−1743	−1461	−1440	−1440	−1438
Chi^2^ statistic	20.55	493.2	531.7	531.8	532.3	43.65	493.9	530.6	530.6	530.6
*p*-value	0.00	0.00	0.00	0.00	0.00	0.00	0.00	0.00	0.00	0.00

Note: Robust standard errors in parentheses. *** *p* < 0.01, ** *p* < 0.05, * *p* < 0.1.

**Table 8:** Probit regression results for drinking behavior with time discount factors by sex.

	Dependent Variable: Daily Drinking			
	Male		Female	
Variable	Direct Measure	Proxy Measure	Direct Measure	Proxy Measure
Hyperbolic_discounting	0.0002		0.1702	
	(0.0988)		(0.1250)	
Procrastination		0.0567 **		0.0453
		(0.0275)		(0.0337)
Sign_effect	0.0478	0.0468	0.2004 **	0.2118 **
	(0.0776)	(0.0778)	(0.0984)	(0.0979)
Impatience	0.0218	0.0153	0.0929	0.1007
	(0.0515)	(0.0515)	(0.0698)	(0.0692)
Age	0.1070 ***	0.1082 ***	0.0768 ***	0.0758 **
	(0.0244)	(0.0246)	(0.0297)	(0.0297)
Age squared	−0.0009 ***	−0.0009 ***	−0.0008 ***	−0.0008 **
	(0.0002)	(0.0002)	(0.0003)	(0.0003)
University	−0.0469	−0.0333	0.0510	0.0697
	(0.0782)	(0.0784)	(0.1243)	(0.1245)
Married	0.1524	0.1613	0.3519 **	0.3507 **
	(0.1577)	(0.1583)	(0.1678)	(0.1679)
Divorced	0.1981	0.2007	0.4520 ***	0.4580 ***
	(0.1386)	(0.1392)	(0.1645)	(0.1652)
Household_num	0.0093	0.0078	0.0288	0.0288
	(0.0288)	(0.0288)	(0.0354)	(0.0356)
Children	0.3505 **	0.3719 **	−0.1406	−0.1353
	(0.1438)	(0.1450)	(0.1757)	(0.1742)
Unemployed	−0.1029	−0.0942	−0.1991 **	−0.2016 **
	(0.1163)	(0.1165)	(0.0955)	(0.0956)
Log_hincome	0.0975	0.0902	−0.1349	−0.1368
	(0.0696)	(0.0697)	(0.0839)	(0.0838)
Log_hasset	−0.0539	−0.0493	0.0626	0.0630
	(0.0397)	(0.0398)	(0.0495)	(0.0490)
Smoking	0.3580 ***	0.3537 ***	0.5880 ***	0.5752 ***
	(0.0754)	(0.0755)	(0.1177)	(0.1179)
Gambling	−0.0465	−0.0452	0.2188	0.2127
	(0.1061)	(0.1066)	(0.2430)	(0.2449)
Risk aversion	0.0395	0.0644	−0.2246	−0.1830
	(0.1791)	(0.1786)	(0.2212)	(0.2223)
Happiness	0.0174	0.0179	0.0310	0.0309
	(0.0217)	(0.0217)	(0.0265)	(0.0266)
Health anxiety	−0.0715	−0.0697	0.0268	0.0286
	(0.0721)	(0.0722)	(0.0882)	(0.0883)
Constant	−4.4521 ***	−4.6730 ***	−2.4978 *	−2.6079 **
	(1.1047)	(1.1091)	(1.3093)	(1.3102)
Obs.	1388	1388	1605	1605
Log likelihood	−881.1	−879	−536.9	−536.9
Chi^2^ statistic	140.1	144	66.99	67.17
*p*-value	0.00	0.00	0.00	0.00

Note: Robust standard errors in parentheses. *** *p* < 0.01, ** *p* < 0.05, * *p* < 0.1.

**Table 9:** Probit regression results for drinking behavior with time discount factors by age group.

	Dependent Variable: Daily Drinking					
	Under 40 Years		40–60 Years		Over 60 Years	
Variable	Direct Measure	Proxy Measure	Direct Measure	Proxy Measure	Direct Measure	Proxy Measure
Hyperbolic_discounting	0.2761 *		0.0866		−0.1067	
	(0.1604)		(0.1094)		(0.1632)	
Procrastination		0.0268		0.0578 *		0.0808 *
		(0.0409)		(0.0304)		(0.0448)
Sign_effect	0.1809	0.1890	0.1322	0.1386	−0.0017	−0.0043
	(0.1295)	(0.1295)	(0.0857)	(0.0856)	(0.1118)	(0.1122)
Impatience	0.0233	0.0327	0.0803	0.0843	0.0359	0.0289
	(0.0929)	(0.0923)	(0.0558)	(0.0556)	(0.0877)	(0.0882)
Male	0.4653 ***	0.4635 ***	1.1200 ***	1.1024 ***	1.2946 ***	1.2617 ***
	(0.1296)	(0.1300)	(0.0918)	(0.0923)	(0.1295)	(0.1314)
Age	−0.1389	−0.1518	0.0760	0.0692	0.0329	0.0490
	(0.1414)	(0.1410)	(0.1335)	(0.1336)	(0.4596)	(0.4664)
Age squared	0.0027	0.0029	−0.0006	−0.0006	−0.0003	−0.0004
	(0.0022)	(0.0022)	(0.0013)	(0.0013)	(0.0034)	(0.0035)
University	−0.0085	0.0177	0.0181	0.0369	−0.1241	−0.1147
	(0.1283)	(0.1290)	(0.0917)	(0.0917)	(0.1494)	(0.1501)
Married	0.3121	0.3257	0.2947	0.2896	−0.1775	−0.1642
	(0.2192)	(0.2166)	(0.1795)	(0.1793)	(0.2460)	(0.2488)
Divorced	−0.0147	−0.0103	0.4821 ***	0.4815 ***	0.0036	0.0242
	(0.2428)	(0.2411)	(0.1587)	(0.1582)	(0.2145)	(0.2184)
Household_num	−0.0111	−0.0135	0.0246	0.0252	0.0423	0.0427
	(0.0448)	(0.0452)	(0.0329)	(0.0329)	(0.0460)	(0.0463)
Children	0.3898 *	0.3783 *	−0.0895	−0.0661	0.2721	0.3106
	(0.2080)	(0.2055)	(0.1705)	(0.1714)	(0.2596)	(0.2636)
Unemployed	−0.4903 ***	−0.4922 ***	−0.1489	−0.1541	−0.1064	−0.1032
	(0.1578)	(0.1568)	(0.1236)	(0.1238)	(0.1178)	(0.1181)
Log_hincome	−0.0818	−0.0906	0.1254	0.1161	−0.1455	−0.1620
	(0.1160)	(0.1159)	(0.0801)	(0.0797)	(0.1036)	(0.1035)
Log_hasset	0.0358	0.0390	−0.0320	−0.0279	0.0135	0.0215
	(0.0756)	(0.0763)	(0.0426)	(0.0425)	(0.0573)	(0.0572)
Smoking	0.4358 ***	0.4285 ***	0.4086 ***	0.4033 ***	0.3912 ***	0.3869 ***
	(0.1252)	(0.1263)	(0.0918)	(0.0917)	(0.1372)	(0.1376)
Gambling	0.1460	0.1373	−0.1640	−0.1701	0.2908	0.2817
	(0.1992)	(0.1987)	(0.1359)	(0.1356)	(0.2169)	(0.2194)
Risk aversion	−0.3538	−0.3216	−0.1665	−0.1236	0.2912	0.3108
	(0.2755)	(0.2726)	(0.1989)	(0.1980)	(0.2902)	(0.2933)
Happiness	−0.0186	−0.0171	0.0355	0.0380	0.0285	0.0282
	(0.0317)	(0.0315)	(0.0236)	(0.0237)	(0.0344)	(0.0346)
Health anxiety	−0.0404	−0.0294	0.0249	0.0230	−0.1333	−0.1350
	(0.1215)	(0.1211)	(0.0774)	(0.0773)	(0.1102)	(0.1106)
Constant	0.6951	0.8890	−5.3629	−5.3364	−0.8463	−1.6179
	(2.6838)	(2.6772)	(3.4918)	(3.5000)	(15.5051)	(15.7394)
Obs.	825	825	1425	1425	743	743
Log likelihood	−330.1	−331.4	−726	−724.4	−354.9	−353.4
Chi^2^ statistic	106.2	104.5	310.3	309.9	168.4	173.4
*p*-value	0.00	0.00	0.00	0.00	0.00	0.00

Note: Robust standard errors in parentheses. *** *p* < 0.01, * *p* < 0.1.

## Data Availability

Data are available upon request.

## Appendix A

**Table A1:** Question 6. You have two options to receive some money. You may choose Option “A” to receive JPY 10,000 two days from today, or Option “B” to receive a different amount nine days from today. Compare the amounts and timings in Options “A” and “B” and indicate which amount you would prefer to receive for each of the following eight choices.

Option “A”: Receive in 2 Days	Option “B”: Receive in 9 Days	Includes an Annual Interest Rate of:	Circle “A” or “B”	
JPY 10,000	JPY 9,981	−10%	A	B
JPY 10,000	JPY 10,000	0%	A	B
JPY 10,000	JPY 10,019	10%	A	B
JPY 10,000	JPY 10,038	20%	A	B
JPY 10,000	JPY 10,096	50%	A	B
JPY 10,000	JPY 10,191	100%	A	B
JPY 10,000	JPY 10,383	200%	A	B
JPY 10,000	JPY 10,574	300%	A	B

**Table A2:** Question 7. You have two options to receive some money. You may choose Option “A” to receive JPY 10,000 90 days from today, or Option “B” to receive a different amount 97 days from today. Compare the amounts and timings in Options “A” and “B” and indicate which amount you would prefer to receive for each of the following eight choices.

Option “A”: Receive in 90 Days	Option “B”: Receive in 97 Days	Includes an Annual Interest Rate of:	Circle “A” or “B”	
JPY 10,000	JPY 9,981	−10%	A	B
JPY 10,000	JPY 10,000	0%	A	B
JPY 10,000	JPY 10,019	10%	A	B
JPY 10,000	JPY 10,038	20%	A	B
JPY 10,000	JPY 10,096	50%	A	B
JPY 10,000	JPY 10,191	100%	A	B
JPY 10,000	JPY 10,383	200%	A	B
JPY 10,000	JPY 10,574	300%	A	B

**Table A3:** Question 8. You have the option to receive JPY 10,000 1 month from today, or receive a different amount 13 months from today. Compare the amounts and timings in Options “A” and “B” and indicate which amount you would prefer to receive for each of the following eight choices.

Option “A”: Receive in One Month	Option “B”: Receive in 13 Months	Includes an Annual Interest Rate of:	Circle “A” or “B”	
JPY 10,000	JPY 9,500	−5%	A	B
JPY 10,000	JPY 10,000	0%	A	B
JPY 10,000	JPY 10,200	2%	A	B
JPY 10,000	JPY 10,400	4%	A	B
JPY 10,000	JPY 10,600	6%	A	B
JPY 10,000	JPY 11,000	10%	A	B
JPY 10,000	JPY 12,000	20%	A	B
JPY 10,000	JPY 14,000	40%	A	B

**Table A4:** Question 9. You have the option to receive JPY 1,000,000 1 month from today, or receive a different amount 13 months from today. Compare the amounts and timings in Options “A” and “B” and indicate which amount you would prefer to receive for each of the following eight choices.

Option “A”: Receive in One Month	Option “B”: Receive in 13 Months	Includes an Annual Interest Rate of:	Circle “A” or “B”	
JPY 1,000,000	JPY 950,000	−5%	A	B
JPY 1,000,000	JPY 1,000,000	0%	A	B
JPY 1,000,000	JPY 1,001,000	0.1%	A	B
JPY 1,000,000	JPY 1,005,000	0.5%	A	B
JPY 1,000,000	JPY 1,010,000	1%	A	B
JPY 1,000,000	JPY 1,020,000	2%	A	B
JPY 1,000,000	JPY 1,060,000	6%	A	B
JPY 1,000,000	JPY 1,100,000	10%	A	B

**Table A5:** Question 10. You have the option to pay JPY 1,000,000 1 month from today or pay a different amount 13 months from today. Compare the amounts and timings in Options “A” and “B” and indicate which amount you would prefer to receive for each of the following eight choices.

Option “A”: Pay in One Month	Option “B”: Pay in 13 Months	Includes an Annual Interest Rate of:	Circle “A” or “B”	
JPY 1,000,000	JPY 950,000	−5%	A	B
JPY 1,000,000	JPY 1,000,000	0%	A	B
JPY 1,000,000	JPY 1,001,000	0.1%	A	B
JPY 1,000,000	JPY 1,005,000	0.5%	A	B
JPY 1,000,000	JPY 1,010,000	1%	A	B
JPY 1,000,000	JPY 1,020,000	2%	A	B
JPY 1,000,000	JPY 1,060,000	6%	A	B
JPY 1,000,000	JPY 1,100,000	10%	A	B
